# Effect of the STK11 mutation on therapeutic efficacy and prognosis in patients with non-small cell lung cancer: a comprehensive study based on meta-analyses and bioinformatics analyses

**DOI:** 10.1186/s12885-024-12130-y

**Published:** 2024-04-17

**Authors:** Ke Xu, Weinan Lu, Airu Yu, Hongwei Wu, Jie He

**Affiliations:** 1 Clinical Medical College, Chengdu Medical College, Chengdu, Sichuan China; 2 Department of Oncology, The First Affiliated Hospital of Chengdu Medical College, Chengdu, Sichuan China; 3 Department of Gastroenterology, The First Affiliated Hospital of Chengdu Medical College, Chengdu, Sichuan China; 4 Department of Pulmonary and Critical Care Medicine, The First Affiliated Hospital of Chengdu Medical College, Chengdu, Sichuan China

**Keywords:** STK11 mutation, Non-small cell lung cancer, Efficacy, Prognosis, Meta-analysis

## Abstract

**Background:**

This study aimed to systematically analyze the effect of a serine/threonine kinase (STK11) mutation (STK11^mut^) on therapeutic efficacy and prognosis in patients with non-small cell lung cancer (NSCLC).

**Methods:**

Candidate articles were identified through a search of relevant literature published on or before April 1, 2023, in PubMed, Embase, Cochrane Library, CNKI and Wanfang databases. The extracted and analyzed data included the hazard ratios (HRs) of PFS and OS, the objective response rate (ORR) of immune checkpoint inhibitors (ICIs), and the positive rates of PD-L1 expression. The HR of PFS and OS and the merged ratios were calculated using a meta-analysis. The correlation between STK11^mut^ and clinical characteristics was further analyzed in NSCLC datasets from public databases.

**Results:**

Fourteen retrospective studies including 4317 patients with NSCLC of whom 605 had STK11^mut^ were included. The meta-analysis revealed that the ORR of ICIs in patients with STK11^mut^ was 10.1% (95%CI 0.9–25.2), and the positive rate of PD-L1 expression was 41.1% (95%CI 25.3–57.0). STK11^mut^ was associated with poor PFS (HR = 1.49, 95%CI 1.28–1.74) and poor OS (HR = 1.44, 95%CI 1.24–1.67). In the bioinformatics analysis, PFS and OS in patients with STK11 alterations were worse than those in patients without alterations (*p* < 0.001, *p* = 0.002). Nutlin-3a, 5-fluorouracil, and vinorelbine may have better sensitivity in patients with STK11^mut^ than in those with STK11^wt^.

**Conclusions:**

Patients with STK11-mutant NSCLC had low PD-L1 expression and ORR to ICIs, and their PFS and OS were worse than patients with STK11^wt^ after comprehensive treatment. In the future, more reasonable systematic treatments should be explored for this subgroup of patients with STK11-mutant NSCLC.

**Supplementary Information:**

The online version contains supplementary material available at 10.1186/s12885-024-12130-y.

## Background

Approximately 1.8 million people die from lung cancer worldwide every year, and 85% of those cases are non-small cell lung cancer (NSCLC) [1, 2]. As a disease of public health importance, rapid advances in early diagnosis method for this disease, as well as the development of drugs for immunotherapy and targeted therapy, have improved patients’ prognosis in recent years [3]. However, further improvement of the overall survival (OS) of patients with NSCLC and a reduction in mortality are required because it is one of the top three causes of malignant tumor-related deaths.

Currently, the exploration of precise and individualized medicine is the core direction of clinical cancer research. In the field of NSCLC, identifying the characteristic subgroups of patients will be the main focus of future work. Therefore, it is essential to explore novel and useful biomarkers and establish reclassifications based on clinical characteristics. On the one hand, this can improve the predictive effect of diagnosis and treatment for NSCLC; on the other hand, it will explore the outcomes of patients who receive newly developed targeted therapy and immunotherapy.

Targeted therapy has been shown to have a conspicuous curative effect. The development of targeted therapies has benefited from breakthroughs in genetic mutation research. Tumor progression resulting from driver gene mutations, such as EGFR and ALK mutations can be prevented by specific targeted drugs [4, 5]. Additionally, some genetic mutations, such as KRAS and KEAP1 mutations, have also been found to be significantly associated with clinical features, therapeutic efficacy, and prognosis [6, 7]. The serine/threonine kinase (STK11) gene, which is located at position 19p13.3, contains 12 exons. The protein encoded by STK11 is a serine/threonine kinase distributed in the nucleus, cytoplasm, and mitochondria. It is also closely associated with the regulation of cell polarity and energy metabolism [8]. Previous studies have shown that it enables the activation of adenosine monophosphate-activated protein kinase (AMPK), which leads to the inhibition of the downstream mTOR signaling pathway [9]. In recent years, it has been found that STK11 has a high rate of mutation in NSCLC, occurring in approximately 15-35% of cases [10]. Researchers initially demonstrated a correlation between STK11 mutations (STK11^mut^) and the prognosis of patients with NSCLC [11]. Subsequently, a significant relationship between STK11^mut^ and immunotherapy efficacy was reported [12, 13]. Over the last three years, some studies have attempted to analyze and interpret the questions arising from the attractive issues highlighted above through retrospective analyses. However, these researchers have not yet reached a consensus. Therefore, this meta-analysis was performed to address these questions, and aims to provide theoretical clues for clinical decision-making.

## Method

### Search strategy

This study involved a comprehensive and systematic search of the PubMed, Embase, Cochrane Library, Wanfang, and CNKI databases. Literature on STK11/LKB1 mutations in patients with NSCLC published on or before April 1, 2023, was searched. Furthermore, the bibliographies of related papers were analyzed to identify papers that did not appear in the database searches. Data were retrieved using a combination of subject headings and free words, including non-small cell lung cancer, non-small cell lung carcinoma, non-small cell lung tumor, lung adenocarcinoma, lung squamous cell carcinoma, STK11 mutation, LKB1 mutation, PJS mutation, and hLKB1 mutation.

### Inclusion and exclusion criteria

#### Inclusion criteria

The following inclusion criteria that guided the study based on the research purpose and design:

1. All patients in the study were diagnosed with NSCLC based on pathological or cytological findings. The extracted clinical characteristics included histology, stage, and PD-L1 status, which was defined by TPS the Tumor cell proportion score (TPS) method (considered positive if ≥ 1%).
2. The types of studies included were observational studies, such as case-control studies, prospective cohort studies or retrospective cohort studies.
3. The included studies were those that reported the STK11 status of the patients. The samples were tested by the next generation sequencing (NGS). The studies should describe and analyze the prognosis or report the response to immune checkpoint inhibitors (ICIs) or PD-L1 expression of the patients.

#### Exclusion criteria

During the screening process, studies that met the following criteria were excluded: (1) studies that did not report on STK11^mut^; (2) studies that did not provide relevant data in the text and those in which the corresponding data could not be obtained by tracing file attachments or contacting the original author; (3) unrelated or repetitive studies; and (4) case reports, conference presentations, abstracts or letters.

### Study screening and quality evaluation

The screening process followed The Preferred Reporting Items for Systematic reviews and Meta-Analyses (PRISMA) statement. For the included cohort and case-control studies, the Newcastle-Ottawa Scale (NOS) scale was used for quality evaluation. This scale evaluates the study using multiple items based on three aspects: selection of subjects, exposure/outcome, and comparability. On this scale, studies are scored on a scale of 0–9 points. When the score is greater than 6 points, the study is considered high-quality.

### Data extraction and processing

All the researchers performed independent literature screening and data extraction. After obviously irrelevant literature was excluded, the full text of the remaining papers was read to determine whether they should be included in the study. The data extracted included the following: (1) basic information included in the research, such as corresponding author, publication year, ethnicity, tumor stage, treatment, the total number of patients, and the number of patients with the STK11 mutation; (2) the primary outcome measure was the hazard ratio (HR) of the OS in multivariate analyses, and the secondary outcome indicators included the HR of progression-free survival (PFS) in multivariate analyses, the objective response rate (ORR) to ICIs, and the positive rate of PD-L1 expression.

### Bioinformatic analysis

Multiple datasets of NSCLC, lung adenocarcinoma (LUAD), and lung squamous cell carcinoma (LUSC) were analyzed using the cBioPortal database, and STK11^mut^ patient information was analyzed to compare therapeutic efficacy and prognosis between patients with STK11^mut^ and those with STK11^wt^. Furthermore, the Genomics of Drug Sensitivity in Cancer (GDSC) database was used to analyze the differences in drug sensitivity between patients with STK11^mut^ and those with STK11^wt^.

### Statistical analysis

STATA11.0 statistical software (http://www.stata.com), was used for data pooling. The HRs and its 95% CIs from each study were used to calculate the pooled HR and 95% CI. A meta-analysis was also conducted on the response rate to immunotherapy and the positive rate of PD-L1 expression. Statistical significance was set at *p* < 0.05. Heterogeneity among the results of the included studies was tested (α = 0.1). I^2^ represented the magnitude of the heterogeneity. If there was no heterogeneity between the results of various studies (*p* > 0.1 or I^2^ < 50%), a fixed effects model was used for the meta-analysis. If there was significant heterogeneity between the research results (*p* ≤ 0.1 or I^2^ > 50%), the source of heterogeneity was first identified. A subgroup analysis was performed for patients with significant heterogeneity in clinical characteristics. For those without significant heterogeneity in clinical characteristics, a random effects model was used for the meta-analysis. The Egger’s bias test and Begg’s funnel plot were used to evaluate publication bias.

## Result

### Literature inclusion and quality assessment

**Fig. 1:**
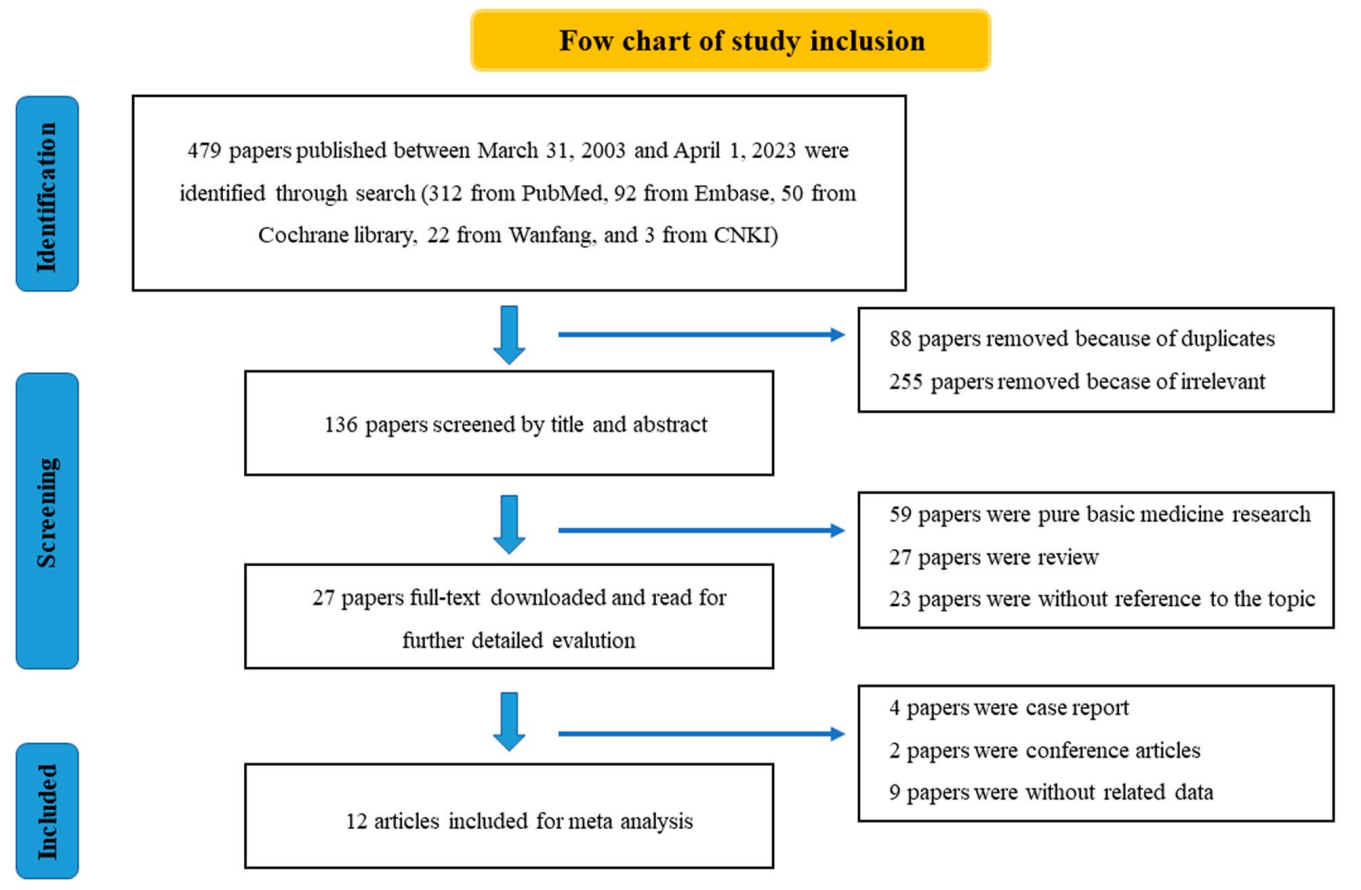
Detailed process of literature identification and screening

A total of 479 papers were retrieved using the search terms. After excluding irrelevant studies, duplicates, pure basic experimental studies, and other unqualified studies, 12 pieces of literature were included, including 14 retrospective studies, among which two papers of Ascierto ML and Shire NJ contained two studies respectively [14–21, 12, 22–26]. A flow chart of the study inclusion is shown in Fig. 1. Subsequently, a quality assessment of the remaining records was conducted using the NOS. The final NOS scores of the 14 studies were as follows: 9 points for 6 studies, 8 points for 6 studies, and 7 points for 2 studies. Further details are presented in Table S1.

### Data extraction

The 14 studies included 4317 patients, including 605 patients with STK11^mut^. All 14 included studies were retrospective studies. Nine studies were conducted in Caucasian regions and five in non-Caucasian regions. Ten studies included patients who received ICIs. Four studies were studies that included patients who did not receive ICIs or those in which ICI therapy was not explicitly mentioned. Six studies presented the PFS, seven presented the OS, six provided the ORR to ICIs, five provided data on PD-L1 expression in patients with the STK11 mutation, and four provided data on PD-L1 expression in patients with STK11^wt^. Detailed information is provided in Table 1.

**Table 1:** Information on the clinical trials eligible for enrollment and their specific characteristics

Author	Year	Country	Trial design	Total patients	STK11^mut^ patients	Patients with KRAS^mut^ and STK11^mut^	Patients with KRAS^mut^ and STK11^wt^	Stage	Treatment	Multivariable HR of PFS	Multivariable HR of OS	Total STK11^mut^ patients treated with ICIs	CR/PR STK11^mut^ patients treated with ICIs	Total STK11^wt^ patients treated with ICIs	CR/PR STK11^wt^ patients treated with ICIs	Total STK11^mut^ patients detected PD-L1	STK11^mut^ patients with PD-L1 ≥ 50%	STK11^mut^ patients with PD-L1 ≥ 1% and < 50%	STK11^mut^ patients with PD-L1 < 1%	Total STK11^wt^ patients detected PD-L1	STK11^wt^ patients with PD-L1 ≥ 50%	STK11^wt^ patients with PD-L1 ≥ 1% and < 50%	STK11^wt^ patients with PD-L1 < 1%	NOS score
Yoh K	2021	Japan	RA	832	52	11	NA	II to IV or recurrent	CT, ICIs	NA	NA	18	0	242	19	52	9	21	22	NA	NA	NA	NA	7
Facchinetti F	2017	France	RA	302	25	13	NA	II to IV	S, CT, RT	1.33 (95%: CI: 0.83–2.12)	1.31 (95% CI: 0.80–2.14)	NA	NA	NA	NA	NA	NA	NA	NA	NA	NA	NA	NA	8
Xu M	2021	China	RA	447	27	NA	NA	IA-IIIB	S, CT	NA	1.046 (95% CI: 0.6975–1.256)	NA	NA	NA	NA	NA	NA	NA	NA	NA	NA	NA	NA	8
Abu Hejleh T	2021	USA	RA	70	11	3	11	III	S, CT, RT, ICIs	2.25 (95% CI: 1.03–4.88)	NA	NA	NA	NA	NA	8	1	2	5	32	10	6	16	9
Ascierto ML (a)*	2021	USA	RA	181	17	10	47	III/IV	CT, ICIs	NA	NA	17	1	164	34	NA	NA	NA	NA	NA	NA	NA	NA	9
Ascierto ML (b)*	2021	USA	RA	121	26	14	24	III/IV	CT, ICIs	NA	NA	26	1	95	19	NA	NA	NA	NA	NA	NA	NA	NA	9
Shire NJ (c)*	2020	USA	RA	270	40	17	NA	IV	ICIs, CT	NA	NA	34	14	200	89	NA	NA	NA	NA	NA	NA	NA	NA	8
Shire NJ (d)*	2020	USA	RA	670	111	56	NA	IV	ICIs, CT	NA	NA	73	18	420	143	NA	NA	NA	NA	NA	NA	NA	NA	8
Cardona AF	2022	Colombia	RA	204	90	36	35	advanced	ICIs, CT	1.31 (95%CI: 1.12–1.88]	1.33 (95%CI: 1.13–2.21)	NA	NA	NA	NA	NA	NA	NA	NA	NA	NA	NA	NA	8
Heymach JV	2021	USA	RA	164	12	7	44	III	definitive RT, CT	2.53 (95%CI: 1.375–4.657)	2.198 (95%CI: 1.097–4.405)	NA	NA	NA	NA	NA	NA	NA	NA	NA	NA	NA	NA	8
Albacker LA	2018	USA	RA	174	54	NA	NA	IV	ICIs, CT	NA	NA	11	0	55	19	54	11		43	120	55		65	9
Wang H	2021	China	RA	598	60	NA	NA	IIIB/IV	ICIs, CT	1.391 (95%CI: 1.048–1.845)	1.567; (95%CI: 1.160–2.119)	NA	NA	NA	NA	60	5	17	38	538	85	208	255	9
Hong YC	2022	China	RA	125	43	NA	NA	I/II/III	S, CT	NA	2.314 (95%CI: 1.248–4.290)	NA	NA	NA	NA	NA	NA	NA	NA	NA	NA	NA	NA	7
Girodet PO	2022	France	RA	159	37	24	49	Locally advanced /metastatic disease	ICIs, CT	1.87 (95% CI: 1.21–2.89)	2.26 (95% CI: 1.35–3.79)	NA	NA	NA	NA	37	8	12	17	120	46	35	39	9

NA, Not Available; HR, Hazard Ratio; CR, Complete Response; PR, Partial Response; PFS, Progression-Free Survival; OS, Overall Survival; ICIs, Immune Checkpoint Inhibitors; NOS, Newcastle-Ottawa Scale; RA, Retrospective Analysis; S, Surgery; CT, Chemotherapy; RT, Radiation Therapy

(a)* Data from Study 1108 and ATLANTIC; (b)* Data from Study 006; (c)* Data from Flatiron Clinico-Genomic database, and ICIs was used in the 1st line treatment; (d)* Data from Flatiron Clinico-Genomic database, and ICIs was used in the 2nd line treatment

### The relationship between STK11^mut^ and PD-L1 expression, ORR to ICIs, PFS, and OS

**Fig. 2:**
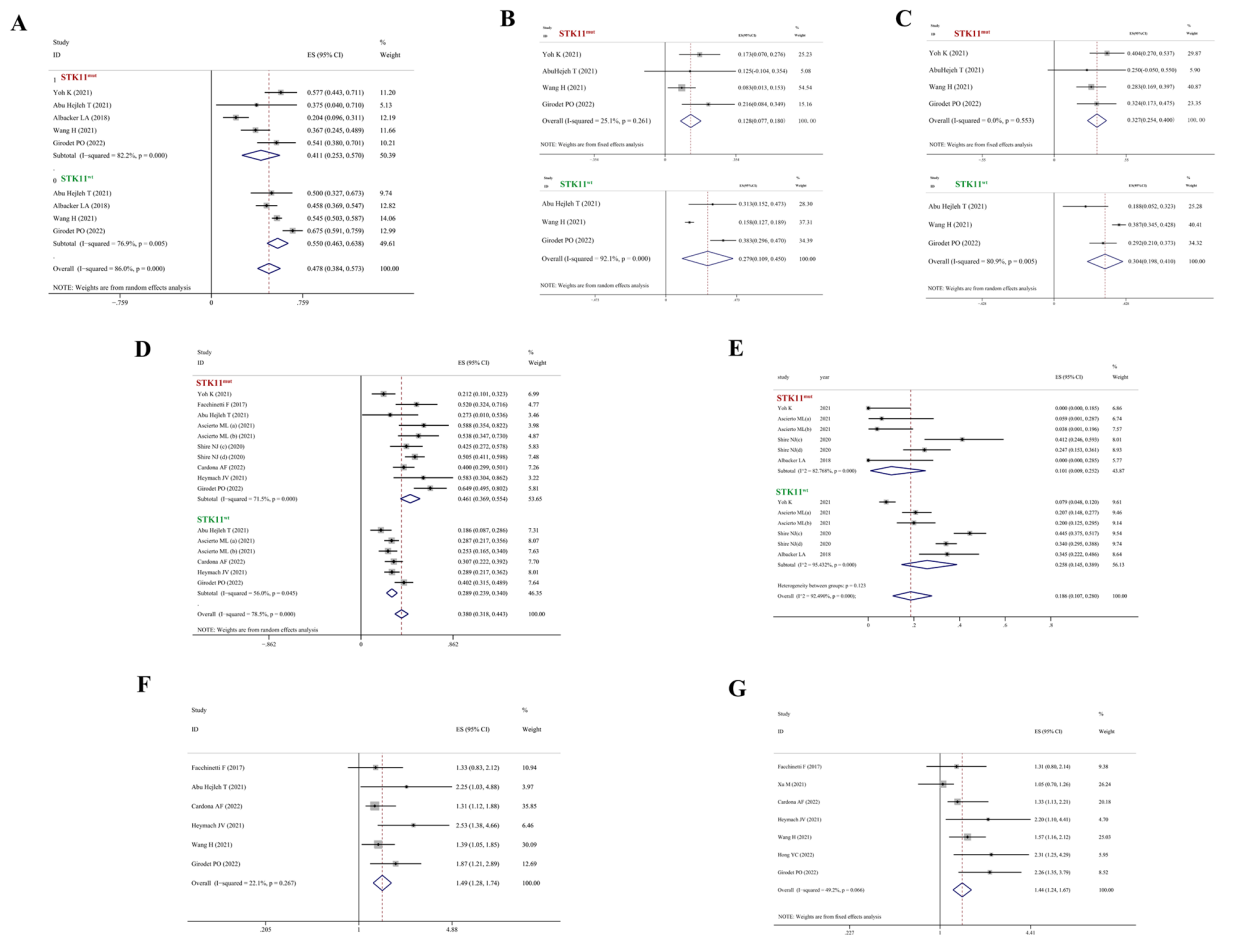
Comparison of PD-L1 expression, efficacy and prognosis between STK11^mut^ and STK11^wt^ patients with NSCLC. (**A**) Overall positive rate of PD-L1 expression. (**B**) Percentage of patients with PD-L1 expression ≥ 50%. (**C**) Percentage of patients with PD-L1 expression ≥ 1% and < 50%. (**D**) Rate of patients with KRAS mutation. (**E**) ORR of patients who received ICIs. (**F**) HR of progressive disease. (**G**) HR of death

For PD-L1 expression, the positivity rates and differences in levels of positivity were investigated. The overall positive rate of PD-L1 expression was 41.1% (95% CI 25.3–57.0) and 55.0% (95% CI 46.3–63.8) in patients with STK11^mut^ and those with STK11^wt^, respectively (Fig. 2A). The strong positive rate (PD-L1 ≥ 50%) was 12.8% (95% CI 7.7–18.0) and 27.9% (95% CI 10.9–45.0) in patients with STK11^mut^ and those with STK11^wt^, respectively (Fig. 2B). The proportion of patients with PD-L1 < 50% and PD-L1 ≥ 1% was 32.7% (95% CI 25.4–40.0), and 30.4% (95% CI 19.8–41.0) in patients with STK11^mut^ and those with STK11^wt^, respectively (Fig. 2C). Another interesting result in the comparison was that the proportion of patients with KRAS mutation was 46.1% (95% CI 36.9–55.4) and 28.9% (95% CI 23.9–34.0) in the patients with STK11^mut^ and those with STK11^wt^, respectively (Fig. 2D). For patients who received ICIs, the ORR was 10.1% (95% CI 0.9–25.2) and 25.8% (95% CI 14.5–38.9) in patients with the STK11 mutation and those with wild type STK11, respectively (Fig. 2E). Upon integrating the results of studies that provided the HR of PFS or OS, STK11^mut^ was found to be an independent and significant prognostic factor for PFS and OS in patients with NSCLC. STK11^mut^ was associated with poor PFS (HR = 1.49, 95% CI 1.28–1.74) and poor OS (HR = 1.44, 95% CI 1.24–1.67) (Fig. 2F-G).

### Subgroup analysis, sensitivity analysis, and publication bias analysis

**Fig. 3:**
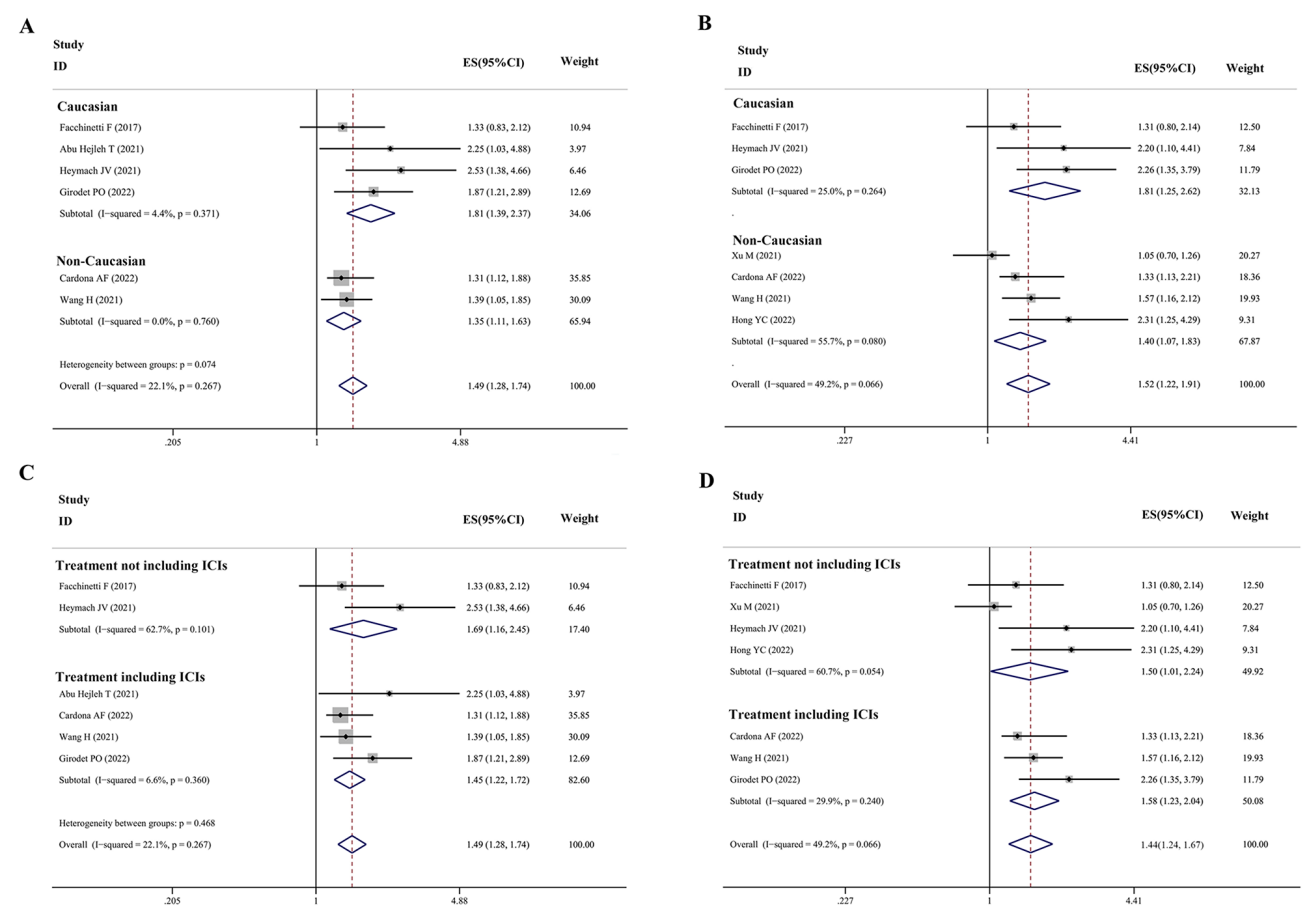
Subgroup analysis for HR of progressive disease and death in STK11^mut^ patients with NSCLC. (**A**) Subgroups were grouped by ethnicity to compare HR of progressive disease. (**B**) Subgroups were grouped by ethnicity to compare the HR of death. (**C**) Subgroups were grouped by whether received ICIs to compare HR of progressive disease. (**B**). Subgroups were grouped by whether received ICIs to compare HR of death

**Fig. 4:**
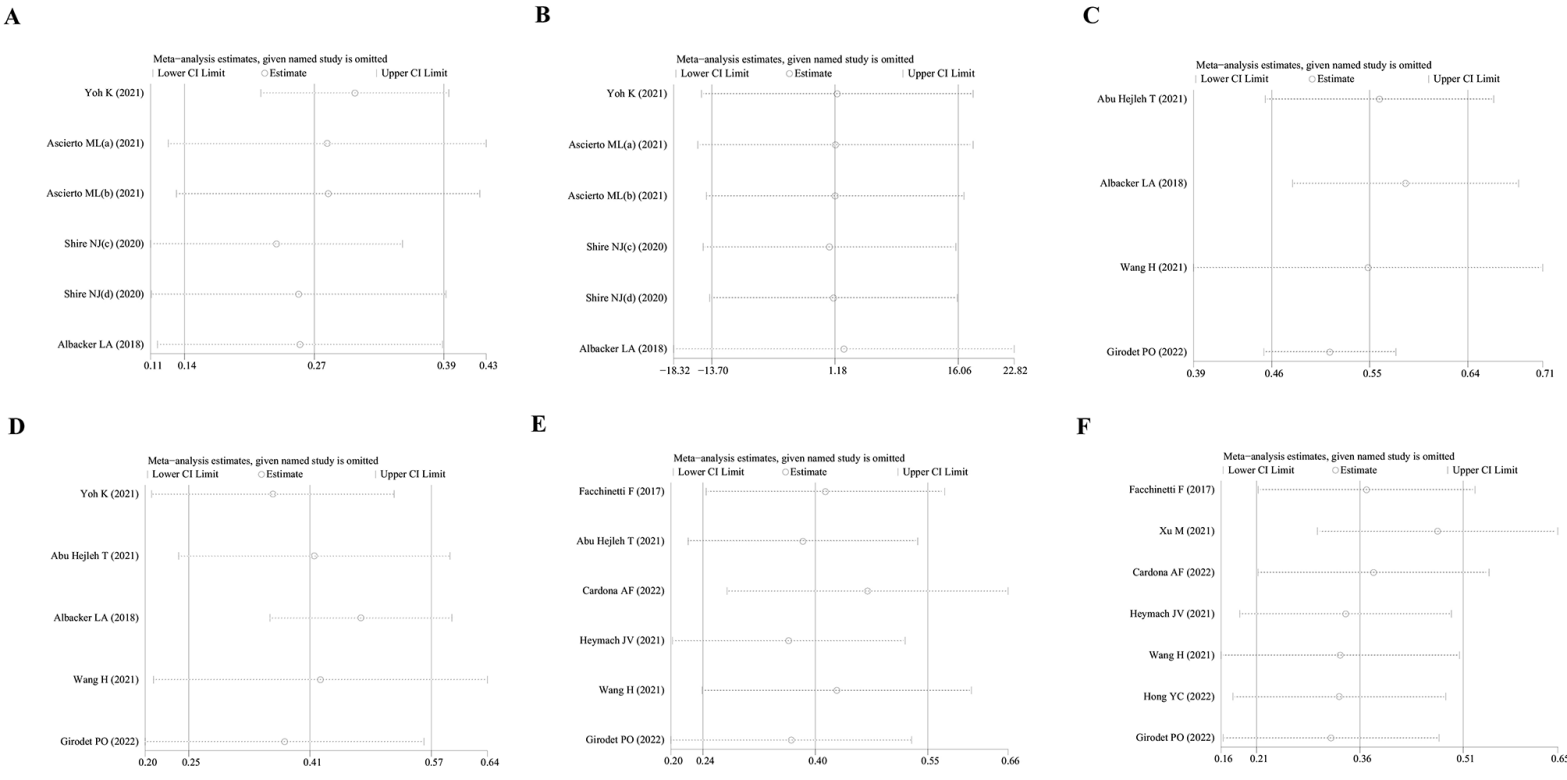
Sensitivity analysis of various observation indicators. (**A**) ORR of STK11^wt^ patients received ICIs. (**B**) ORR of STK11mut patients received ICIs. (**C**) Positive rate of PD-L1 expression in STK11^wt^ patients. (**D**) Positive rate of PD-L1 expression in STK11^mut^ patients. (**E**) HR of progressive disease. (**F**) HR of death

**Fig. 5:**
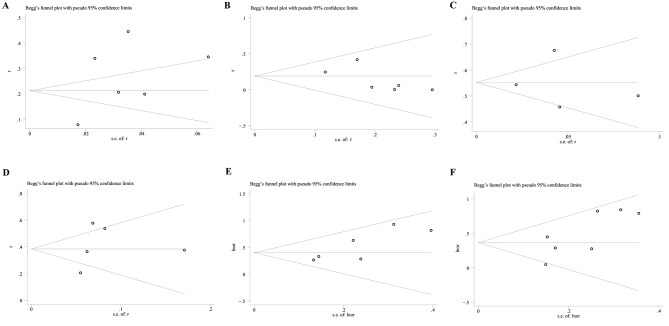
Publication bias analysis of various observation indicators. (**A**) ORR of STK11^wt^ patients received ICIs. (**B**) ORR of STK11^mut^ patients received ICIs. (**C**) Positive rate of PD-L1 expression in STK11^wt^ patients. (**D**) Positive rate of PD-L1 expression in STK11^mut^ patients. (**E**) HR of progressive disease. (**F**) HR of death

In this study, there was no statistical heterogeneity in the HR of PFS and OS. However, we rigorously conducted a subgroup analysis according to ethnicity and treatment with ICIs. Firstly, we found STK11^mut^ was associated with a poor PFS in both Caucasian and non-Caucasian subgroups (HR = 1.81, 95% CI 1.39–2.37 and HR = 1.35, 95% CI 1.11–1.63, respectively) (Fig. 3A). During the comparison, the I^2^ decreased significantly, and heterogeneity was further reduced. Similarly, subgroup analysis indicated that the OS of patients with STK11 mutations from both regions was significantly shortened (HR = 1.81, 95% CI 1.25–2.62 and HR = 1.40, 95% CI 1.07–1.83, respectively) (Fig. 3B). Based on the HR values, the effect of STK11^mut^ on PFS and OS may be higher in Caucasians. Secondly, for patients who were not treated with ICIs, STK11^mut^ was associated with a statistically worse PFS (HR = 1.69, 95% CI 1.16–2.45) and OS (HR = 1.50, 95% CI 1.01–2.24). For patients who received ICIs, the PFS and OS were also shorter among patients with the STK11 mutation (HR = 1.45, 95% CI 1.22–1.72 and HR = 1.58, 95% CI 1.23–2.04, respectively). The effect of STK11^mut^ on the PFS and OS of patients was not significantly different between the group that received ICIs and the group that did not receive ICIs (Fig. 3C-D). Regarding the sensitivity analysis, no study was observed to be the source of heterogeneity; therefore, none were excluded from analyses (Fig. 4A-F). Publication bias was assessed using Egger’s bias test. The *p*-values for the positive rate of PD-L1 expression in patients with STK11^mut^, the positive rate of PD-L1 expression in patients with STK11^wt^, the ORR to ICIs in patients with STK11^mut^, the ORR to ICIs in patients with STK11^wt^, the HR of the PFS, and the OS were 0.561, 0.971, 0.255, 0.159, 0.089, and 0.661, respectively. Based on the above results, no publication bias was found in the included studies (Fig. 5A-F).

### The association between STK11^mut^ and patient clinical characteristics and prognosis in the public data sets

**Fig. 6:**
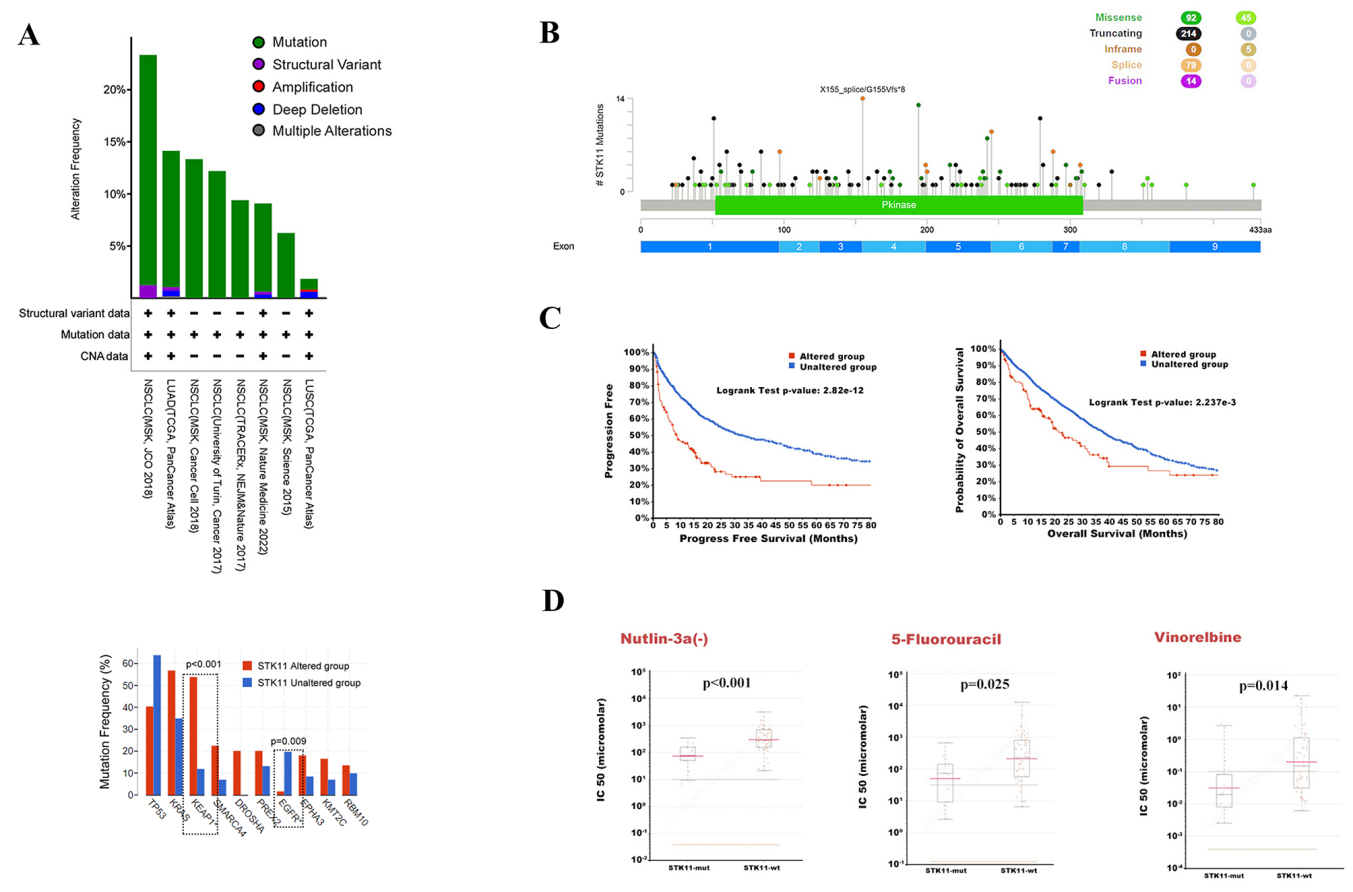
Bioinformatic analysis of clinical characteristics of STK11^mut^ and STK11^wt^ patients with NSCLC. (**A**) STK11 alteration in multiple NSCLC datasets and the co-occurring genetic mutations in STK11 altered and unaltered patients. (**B**) Type and distribution of STK11^mut^ in multiple NSCLC datasets. (**C**) Comparison of PFS and OS between STK11 altered and unaltered patients with NSCLC. (**D**) Comparison of drug sensitivity between STK11^mut^ and STK11^wt^ patients with NSCLC.

STK11 was further analyzed in multiple NSCLC datasets using the cBioPortal database. STK11 alterations were found in 1.85–23.33% of the patients, among which the most common type was a mutation. STK11-mutant patients had a higher rate of concurrent KEAP1 mutation; however, STK11/EGFR mutations were significantly less common (*p* < 0.001, *p* = 0.009) (Fig. 6A-B). In the survival analysis of the above patients, PFS and OS in patients with STK11 alterations were worse than those in patients without STK11 alterations, which is consistent with the results of the meta-analysis (Fig. 6C). Finally, the GDSC database was used to predict drug sensitivity in patients with NSCLC and STK11^mut^, Nutlin-3a, 5-Fluorouracil, and vinorelbine had better sensitivity in patients with STK11^mut^ than in those with STK11^wt^ (*p* < 0.001, *p* = 0.025, *p* = 0.014) (Fig. 6D).

## Discussion

STK11^mut^ is the second most common mutation in NSCLC, and it often occurs concurrently with the KRAS and KEAP1 mutations [15, 25, 26]. Previous research revealed that STK11 alteration was associated with tumor differentiation, invasion, early lymph node metastasis and TNM stage [27]. Therefore, patients with STK11^mut^ were once considered a special subgroup of patients with NSCLC. Interestingly, it was proved that STK11^mut^ may be closely associated with the efficacy of PD-1/PD-L1 immunological therapy. Researchers initially investigated the relationship between STK11 expression and the tumor immune microenvironment and found that the loss of STK11 expression may result in a reduction in the infiltration of the tumor microenvironment by cytotoxic CD8^+^ T-cells, a decreasing in the expression of STING, and a decline in immune activity. Moreover, it induces high-density neutrophil enrichment by secreting the IL-8 family of cytokines and recruits several myeloid-derived suppressor cells by producing chemokines, such as IL-6, eventually causing the formation of a cold tumor immune microenvironment [18, 26, 28]. The above findings partly explain the poor efficacy of PD-1/PD-L1 drugs in patients with STK11^mut^. Moreover, some clinical researchers demonstrated a significant correlation between STK11 expression and PD-L1 expression in tumor tissues, often showing a lower expression of PD-L1 in patients with STK11^mut^, providing further evidence that STK11^mut^ may indicate poor immunotherapeutic outcomes [22]. Nevertheless, a contradictory conclusion has also been reached. In a retrospective analysis of KEYNOTE-042, researchers discovered that STK11-mutant patients had a superior response to ICIs, which was contrary to the preceding consensus. Some researchers considered that this inverse result may be attributable to the small sample size of the study, which included only 16 patients with STK11^mut^ [29]. To analyze and explain this controversy, we calculated the ORR to ICIs in 179 patients with STK11^mut^ and obtained a result of 10.1% (95%CI 0.9–25.2), while that in 1176 patients with STK11^wt^ was 25.8% (95%CI 14.5.0-38.9). The positive rate of PD-L1 expression was 38.5% (95%CI 18.4–58.6) in 145 STK11-mutant patients, and 51.4% (95%CI 45.4–57.4) in 583 STK11^wt^ patients. These results suggested that patients with STK11^mut^ have a lower positive rate of PD-L1 expression and a limited response to ICIs. Considering that co-mutation of STK11 and KRAS is common, the rate of KRAS mutations was calculated in patients with STK11^mut^ and STK11^wt^. After the pooled effect size was evaluated, the proportion of patients with KRAS mutation was 46.1% (95%CI 36.9–55.4) in the STK11^mut^ patients, and 28.9% (95%CI 23.9–34.0) in the STK11^wt^ patients, respectively. There seemed to be a high rate of KRAS mutation in patients with STK11 mutations. This was consistent with the results of previous studies.

The prediction of the prognosis could influence the design of treatment strategies, therapy options, and follow-up methods after treatment. Relying solely on traditional TNM staging can no longer meet the demands of current precision medicine. Immunohistochemistry, fluorescence in situ hybridization, and gene sequencing technologies have been widely used to recognize the biological characteristics of tumors and are regarded as auxiliary measures for a more accurate prognostic judgment of the disease by clinicians. Presently, with the widespread use of numerous new drugs and the wide application of comprehensive treatment strategies, the overall patient prognosis has significantly improved, and analyses of prognosis and relevant factors have become more complicated. From the perspective of genetic mutation, many studies have proven the significant relationship between genetic mutations and prognosis. For instance, the most common KRAS mutation may indicate a poor prognosis. However, in terms of whether STK11 is related to prognosis, not all existing studies have obtained definitive results. This study demonstrated a shorter OS of STK11-mutant patients compared with the patients with STK11^wt^ based on a meta-analysis of 1999 patients with NSCLC. The PFS after comprehensive therapy also seemed shorter in patients with STK11^mut^. The aforementioned results reached the consistent conclusion that STK11^mut^ was associated with worse outcome after treatment. However, it should be noted that for patients who received ICIs, PD-L1, an indicator of immunotherapy response, was not included in the univariate and multivariate analyses in all studies, which raises the question of whether the inclusion of both factors (STK11 mutation or wild-type STK11and PD-L1 expression level) in the univariate and multivariate analyses would affect the overall results. Unfortunately, because all the included studies only provided data on STK11 mutations and efficacy, it was not possible to extract complete data to answer this question. In the future, determining whether STK11 mutations and PD-L1 expression have a synergistic effect on the prognosis of this subgroup of patients will be necessary. In the context of unsatisfactory outcomes, whether further subclassification is required and whether the combined application of targeted drugs is needed are novel problems worth further exploration in the future.

Regarding the treatment of patients with STK11 mutations, no specific targeted drug s are available for the treatment of patients with STK11 mutations. Recently, treatment exploration for this population has gradually been launched. Preclinical studies have found that STK11 deficient cell lines are more sensitive to metformin [30]. A Phase II clinical study using metformin, pemetrexed, and carboplatin for the treatment of STK11-mutated NSCLC did not demonstrate the expected efficacy (CTRI/2019/02/0017815) [31]. Recently, another clinical trial used talazoparib plus avelumab to treat stage IV or recurrent non-squamous NSCLC with STK11 mutations. The inclusion criterion was that patients had received at least one line of anti-PD-1/PD-L1 therapy. Finally, 42 eligible patients were enrolled, and the ORR was 4.76%, PFS was 2.7 months (95%CI 1.6–3.9), and OS was 7.6 months (95%CI 6.3–12.2) (NCT04173507) [32]. Clinical trials are currently underway. Crystal et al. planned to treat STK11 mutant solid tumors by combining TNG260 with anti-PD1 therapy. The basis of the design was mainly to reverse PD1 resistance driven by STK11 deletion through TNG260, a CoREST inhibitor (NCT05887492) [33]. Another notable study is using daratumumab to treat patients with STK11-mutant NSCLC who have failed in the standard therapy; this study is still recruiting patients (NCT05807048) [34]. Overall, future studies should focus on overcoming the resistance of STK11 mutation to PD1/PD-L1 immunotherapy. However, the exploration of targeted therapy for signaling pathways to STK11 should also be an important breakthrough direction. In this study, chemotherapeutic drugs that are more sensitive in patients with STK11^mut^ were explored using bioinformatics. Nutlin-3a, 5-fluorouracil, and vinorelbine were found to have better sensitivity. Because Nutlin-3a and 5-fluorouracil are not recommended for the systemic treatment of NSCLC, further investigation is needed to determine whether vinorelbine has better efficacy for in patients with STK11^mut^ in the real world.

In this respect, some specific and in-depth questions are worth discussing. In early-stage NSCLC, the effect of genetic mutations on patient outcomes is a complex and controversial topic. It is currently impossible to conduct an analysis on this topic because related research is still lacking. Additionally, it is difficult to investigate the correlation between STK11^mut^ and the efficacy of specific therapies, mainly because of the wide application of comprehensive treatments for NSCLC. With regard to the response to immunotherapy, this study only analyzed the relationship between STK11^mut^ and the response to PD-1/PD-L1 drugs; however, the relationship between STK11^mut^ and CTLA4, TIGHT, and others is unclear. Additionally, most of the included studies were not grouped to introduce clinical features according to STK11 status, and the baseline characteristics of patients could not be compared, which may have affected the HR analysis of PFS and OS.

## Conclusion

Patients with STK11-mutant NSCLC had low levels of PD-L1 expression and ORR to ICIs, and their PFS and OS were worse than those of patients with STK11^wt^ after comprehensive treatment. In the future, more reasonable systematic treatments should be explored for this subgroup of patients with STK11-mutant NSCLC.

### Electronic supplementary material

Below is the link to the electronic supplementary material.

Supplementary Material 1

## Data Availability

The dataset generated and analyzed during the current study is available in cBioPortal (https://www.cbioportal.org/) and GDSC database (https://www.cancerrxgene.org).

## Abbreviations

STK11: Serine/Threonine Kinase
NSCLC: non-small cell lung cancer
ICIs: Immune Checkpoint Inhibitors
STK11 mutation: STK11^mut^
STK11 wild-type: STK11^wt^
ORR: Objective response rate
PFS: Progression-Free Survival
OS: Overall Survival
HR: Hazard Ratio
AMPK: Adenosine monophosphate-activated protein kinase
NGS: Next Generation Sequencing
NOS: Newcastle-Ottawa Scale
PRISMA: Preferred Reporting Items for Systematic reviews and Meta-Analyses
LUAD: Lung adenocarcinoma,LUSC,lung squamous cell carcinoma
GDSC: Genomics of Drug Sensitivity in Cancer database
NA: Not Available
CR: Complete Response
PR: Partial Response
RA: Retrospective Analysis
S: Surgery
CT: Chemotherapy
RT: Radiation Therapy
