# Supplementary material for: Effect of the STK11 mutation on therapeutic efficacy and prognosis in patients with non-small cell lung cancer: a comprehensive study based on meta-analyses and bioinformatics analyses
Source: BMC Cancer. 2024 Apr 17;24:491. doi: 10.1186/s12885-024-12130-y (PMC11025184; doi:10.1186/s12885-024-12130-y)
Supplement: Supplementary file 1 — Supplementary Material 1 [file 12885_2024_12130_MOESM1_ESM.docx]

Supplementary Table 1: The literature quality scores of the included studies using the Newcastle-Ottawa Scale.

| First author and year | Selection | | | | Comparability | Exposure | | |
| --- | --- | --- | --- | --- | --- | --- | --- | --- |
|  | Q1 | Q2 | Q3 | Q4 | Q1 | Q1 | Q2 | Q3 |
| Yoh K（2021） | * | * |  |  | ** | ** | * |  |
| Facchinetti F（2017） | * | * |  | * | ** | ** | * |  |
| Xu M（2021） | * | * | * |  | ** | ** | * |  |
| Abu Hejleh T（2021） | * | * | * | * | ** | ** | * |  |
| Ascierto ML (A)*（2021） | * | * | * | * | ** | ** | * |  |
| Ascierto ML (B)*（2021） | * | * | * | * | ** | ** | * |  |
| Shire NJ (A)* (2020) | * | * | * |  | ** | ** | * |  |
| Shire NJ (B)* (2020) | * | * | * |  | ** | ** | * |  |
| Cardona AF (2022) | * | * | * |  | ** | ** | * |  |
| Heymach JV (2021) | * | * |  | * | ** | ** | * |  |
| Albacker LA (2018) | * | * | * | * | ** | ** | * |  |
| Wang H (2021) | * | * | * | * | ** | ** | * |  |
| Hong YC (2022) | * | * |  |  | ** | ** | * |  |
| Girodet PO (2022) | * | * | * | * | ** | ** | * |  |

NEWCASTLE - OTTAWA QUALITY ASSESSMENT SCALE

CASE CONTROL STUDIES

Note: A study can be awarded a maximum of one star for each numbered item within the Selection categories. A maximum of two stars can be given for Comparability and Exposure.

Selection

1) Is the case definition adequate?

a) yes, with independent validation *

b) yes, eg record linkage or based on self-reports

c) no description

2) Representativeness of the cases

a) consecutive or obviously representative series of cases *

b) potential for selection biases or not stated

3) Selection of Controls

a) community controls *

b) hospital controls

c) no description

4) Definition of Controls

a) no history of disease (endpoint) *

b) no description of source

Comparability

1) Comparability of cases and controls on the basis of the design or analysis

a) study controls for ______________ (Select the most important factor.) *

b) study controls for any additional factor. (This criteria could be modified to indicate specific control for a second important factor.) *

Exposure

1) Ascertainment of exposure

a) secure record (eg surgical records) *

b) structured interview where blind to case/control status *

c) interview not blinded to case/control status

d) written self-report or medical record only

e) no description

2) Same method of ascertainment for cases and controls

a) yes *

b) no

3) Non-Response rate

a) same rate for both groups *

b) non respondents described

c) rate different and no designation
